# Intra-amniotic *Candida albicans* infection induces mucosal injury and inflammation in the ovine fetal intestine

**DOI:** 10.1038/srep29806

**Published:** 2016-07-14

**Authors:** Maria Nikiforou, Esmee M.R. Jacobs, Matthew W. Kemp, Mathias W. Hornef, Matthew S. Payne, Masatoshi Saito, John P. Newnham, Leon E.W. Janssen, Alan H. Jobe, Suhas G. Kallapur, Boris W. Kramer, Tim G.A.M. Wolfs

**Affiliations:** 1 School for Mental Health and Neuroscience, Maastricht University, Maastricht, the Netherlands; 2 Department of Pediatrics, Maastricht University Medical Center, Maastricht, the Netherlands; 3 School of Women’s and Infants’ Health, The University of Western Australia, Perth, Western Australia; 4 Institute of Medical Microbiology, RWTH University Hospital, Aachen, Germany; 5 Division of Perinatal Medicine, Tohoku University Hospital, Sendai, Japan; 6 Division of Pulmonary Biology, Cincinnati Children’s Hospital Medical Centre, University of Cincinnati School of Medicine, Cincinnati, OH, USA; 7 School of Oncology and Developmental Biology, Maastricht University, Maastricht, the Netherlands; 8 Department of Biomedical Engineering, Maastricht University, Maastricht, the Netherlands

## Abstract

Chorioamnionitis is caused by intrauterine infection with microorganisms including Candida albicans (*C.albicans)*. Chorioamnionitis is associated with postnatal intestinal pathologies including necrotizing enterocolitis. The underlying mechanisms by which intra-amniotic *C.albicans* infection adversely affects the fetal gut remain unknown. Therefore, we assessed whether intra-amniotic *C.albicans* infection would cause intestinal inflammation and mucosal injury in an ovine model. Additionally, we tested whether treatment with the fungistatic fluconazole ameliorated the adverse intestinal outcome of intra-amniotic *C.albicans* infection. Pregnant sheep received intra-amniotic injections with 10^7^ colony-forming units *C.albicans* or saline at 3 or 5 days before preterm delivery at 122 days of gestation. Fetuses were given intra-amniotic and intra-peritoneal fluconazole treatments 2 days after intra-amniotic administration of *C.albicans*. Intra-amniotic *C.albicans* caused intestinal colonization and invasive growth within the fetal gut with mucosal injury and intestinal inflammation, characterized by increased CD3^+^ lymphocytes, MPO^+^ cells and elevated TNF-α and IL-17 mRNA levels. Fluconazole treatment *in utero* decreased intestinal *C.albicans* colonization, mucosal injury but failed to attenuate intestinal inflammation. Intra-amniotic *C.albicans* caused intestinal infection, injury and inflammation. Fluconazole treatment decreased mucosal injury but failed to ameliorate *C.albicans*-mediated mucosal inflammation emphasizing the need to optimize the applied antifungal therapeutic strategy.

Preterm birth, which frequently results from intrauterine infection, represents a major cause of neonatal morbidity and mortality^1^. Chorioamnionitis, defined as inflammation of the chorioamniotic membranes and amniotic fluid^2^, is commonly caused by ascending infection into the uterine cavity^3^,^4^. While numerous different bacterial species have been cultured from the amniotic fluid of affected human fetuses, *Ureaplasma (UP), Fusobacterium and Mycoplasma* are the most commonly isolated microorganisms^2^,^5^,^6^. Microbial growth within the amniotic cavity exposes the fetus to bacterial toxins and inflammatory mediators that cause a fetal inflammatory response syndrome (FIRS) that is associated with postnatal adverse outcomes in multiple fetal organs including the gastrointestinal tract^7^. Preterm infants exposed to chorioamnionitis present in the clinic with a wide spectrum of adverse intestinal consequences ranging from poor nutritional uptake and subsequent postnatal growth deficits^8^ to severe, life-threatening gastrointestinal complications such as necrotizing enterocolitis (NEC)^9^,^10^.

Emerging evidence from recent studies supports an association between fungi (in particular yeasts such as Candida spp.) and chorioamnionitis^11^,^12^,^13^. Although *Candida albicans* (*C.albicans*) can be a commensal organism in the female genital tract, chorioamnionitis with *C.albicans* can cause fetal death or fetal candidiasis with systemic manifestations including sepsis^14^,^15^,^16^. Treatment of *C.albicans* infection during pregnancy has been challenging^17^. Although fluconazole, the most commonly used anti-fungal agent, has been associated with teratogenicity when administered at high doses orally, during the first trimester of pregnancy^18^, clinical cases of *C.albicans*-mediated chorioamnionitis at later stages of gestation were successfully treated with oral and intra-amniotic (IA) fluconazole in the absence of reported adverse effects^15^.

We have previously used a translational ovine chorioamnionitis model and have shown that IA exposure to lipopolysaccharide (LPS), UP or inflammatory mediators induces intestinal inflammation and mucosal injury^19^,^20^,^21^. Amniotic fluid infection with *C.albicans* can result in a reservoir of this organism in the fetal bowel where potentially may induce gastrointestinal pathologies. However, the underlying mechanisms by which intrauterine infection with *C.albicans* may result in adverse intestinal effects remain substantially unexplored. Therefore, we utilized our chorioamnionitis model to assess whether IA *C.albicans* infection caused intestinal colonization, local invasive growth and mucosal intestinal inflammatory responses associated with tissue damage to the fetal gut. Additionally, we tested whether IA and intra-peritoneal fluconazole treatment would ameliorate the adverse effects of *C.albicans*-mediated chorioamnionitis on the fetal ovine gut.

## Materials and Methods

### Animals

All animal experiments were approved by the Animal Ethics Committee of the University of Western Australia (Perth, WA, Australia) and the Children’s Hospital Medical Center (Cincinnati, Ohio, USA). All experimental methods were performed in accordance with the approved guidelines and regulations.

### Experimental design

**Figure 1:**
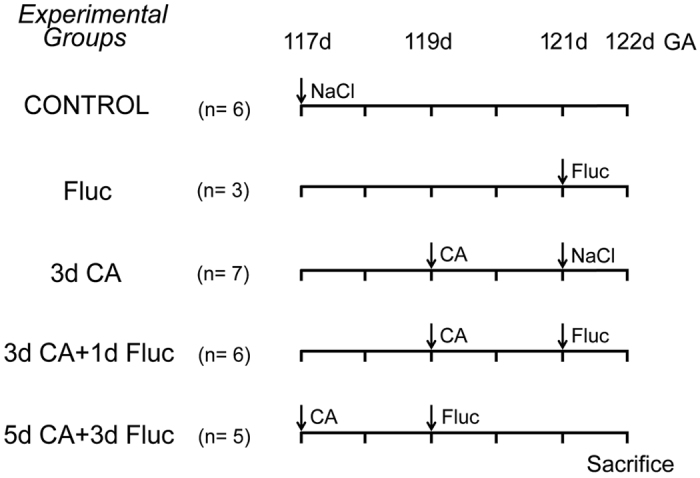
Experimental design. Fetal sheep were given intra-amniotic (IA) *C.albicans* or saline at 3 or 5 days before preterm delivery at 122 days of gestation. IA and intra-peritoneal fluconazole treatment was administered to the fetus 2 days after *C.albicans* infection. CA, *C.albicans*; d, day(s); Fluc, Fluconazole; GA, gestational age; n, number.

All experimental procedures were performed as previously described^22^. Date-mated Merino sheep (*Ovis aries*) were randomly assigned to either 1) a control group (n = 6), which received an IA injection of saline, 2) an antifungal group (n = 3), which received fluconazole treatment only, 3) a 3d *C.albicans* group (n = 7), which received an IA injection of 10^7^ colony-forming units (CFU) of *C.albicans* (all *C. albicans* inoculums prepared in sterile saline) 3 days before preterm delivery and IA injection of saline 1 day before delivery 4) a 3d *C.albicans* and 1d fluconazole group (n = 6), which received an IA injection of 10^7^ CFU *C.albicans* 3 days before preterm delivery and fluconazole treatment 1 day before delivery or 5) a 5d *C.albicans* and 3d fluconazole group (n = 5), which received an IA injection of 10^7^ CFU *C.albicans* 5 days before delivery and fluconazole treatment 3 days before delivery (Fig. 1). Since an IA exposure to *C.albicans* for 5 days was previously shown to result in fetal mortality, our experimental setting does not contain a group of animals only exposed to *C.albicans* for 5 days^22^. A dose of 15 mg fluconazole was injected into the amniotic cavity and 15 mg was given by intra-peritoneal injection to the fetus. This route of administration was chosen because of the long half-life of fluconazole and its direct availability to the fetus through swallowing of the amniotic fluid. All injections were performed under ultrasound guidance. All fetuses were delivered surgically at 122 days (±1 day) of gestation (term at ~147–150 days) simulating a preterm human delivery of approximately 32–34 weeks of gestation.

Fetuses and ewes were euthanized with an intravenous bolus of pentobarbital (100 mg/kg). Fetal terminal ileum was immediately collected, frozen in liquid nitrogen and stored in −80. In addition, fetal terminal ileum was placed in cassettes and immerged in formalin for immunohistochemical stainings.

### Antibodies

The following antibodies were used. Polyclonal rabbit antibody against human CD3 (catalog reference A0452, 1:1000); rabbit antibody against human myeloperoxidase (MPO, catalog reference A0398, 1:500) both from Dako, (Glostrup, Denmark). As secondary antibodies, biotin conjugated swine anti-rabbit (catalog reference E0353, 1:200) from Dako and peroxidase conjugated goat anti-rabbit (catalog reference 111-035-045, 1:200) from Jackson (West Grove, PA) were used.

### Blood cultures

*C.albicans* in blood cultures was detected as described previously^22^. Briefly, fetal blood was inoculated into culture vials and incubated at 37 °C for 72 hours. Every day, 1 ml sample was removed and subcultured on sheep blood agar at 37 °C for 48 hours. *C.albicans* colonial morphology was confirmed by growth on Brilliance Candida Agar (Oxoid, Adelaide, Australia) as previously reported^22^.

### Immunohistochemistry

Formalin-fixed paraffin-embedded fetal terminal ileum was cut in 4 μm thick sections. Morphology was evaluated using hematoxylin-and-eosin (H&E)-staining and fungi were visualized with periodic acid Schiff (PAS) staining. Immunohistochemistry was performed as previously described^23^. After blocking the endogenous peroxidase activity and non-specific binding, sections were incubated with anti-MPO or anti-CD3 antibodies for either 1 hour or overnight, respectively. Thereafter, sections were washed and incubated with a secondary biotin (CD3) or peroxidase (MPO) conjugated antibody. Substrate staining for MPO was performed with 3-amino-9-ethylcarbazole (AEC) from Invitrogen, (Bleiswijk, the Netherlands). CD3 immunoreactivity was detected by using nickel-DAB from Dako, (Glostrup, Denmark). Nuclei were counterstained with either Nuclear Fast Red (CD3) or hematoxylin (MPO). CD3 and MPO expressing cells were counted per high power field (Magnification ×200) and the average number of positive cells in 5 high power fields of terminal ileum per animal is presented. Assessment of the sections was performed as previously described^23^. Briefly, the high power field was defined under the microscope, a picture was captured and subsequently cell counting was performed using Image J.

### Quantitative Real Time PCR (qPCR)

The mRNA expression level of cytokines was measured by qPCR as previously described^24^. Total RNA was isolated from 50 mg freshly frozen ileal tissue by Trizol/chloroform extraction using the SV Total RNA Isolation System (Promega, Madison, WI, USA) according to manufacturer’s guidelines. The samples were treated with RQ1 DNase (Promega, Madison, WI, USA) to eliminate genomic DNA contamination. The extracted RNA was tested for the presence of genomic DNA using ovine specific actin primers by PCR (Westburg Thermocycler, Leusden, the Netherlands) and analyzed with agarose gel electrophoresis. Briefly, PCR amplification was performed with DNA Taq Polymerase (Promega) at 95 °C for 5 minutes, followed by 34 cycles at 95 °C for 30 seconds, 52 °C for 45 seconds and 72 °C for 30 seconds. The quality of PCR products was tested in a 1.2% agarose gel. Reverse transcription was performed with M-MLV Reverse transcriptase from Invitrogen, (Bleiswijk, the Netherlands) according to manufacturer’s guidelines using oligo(dT) primers. qPCR was performed with the SensiMix SYBR & Fluorescein Kit from GC Biotech B.V., (Alphen aan de Den Rijn, the Netherlands) using a LightCycler 480 from Roche Applied Science, (Almere, the Netherlands) according to manufacturer’s instructions. The PCR reactions were run with 10 ng of cDNA, in duplicates using the appropriate primers to specifically amplify the ovine gene transcript (Supplementary Table 1). Based on previous reports^25^,^26^, expression stability and expression level (Cq value ~20) of S15 across several ovine tissues (including the gut) which were tested in our laboratory, we concluded that S15 can serve as a good reference gene. The efficiency of the primers was tested by dilution experiments. The results were normalized to the S15 ribosomal protein (ovRPS15) as a housekeeping gene (with a Cq value of 20), and the mean fold change in mRNA levels relative to the control are presented.

### Enzyme-Linked Immunosorbent Assay (ELISA)

To quantify the degree of infection-induced damage to the intestinal epithelium, the concentration of intestinal fatty acid-binding protein (I-FABP) was measured in fetal plasma samples by ELISA as previously described^27^. Briefly, an Elisa plate was coated with 3 μg/well of anti-human I-FABP monoclonal antibody for 24 hours. A dilution series of a known concentration of human recombinant I-FABP protein was used for a standard curve. Plasma samples were diluted two times and were incubated with biotin-conjugated polyclonal anti-human I-FABP antibody, followed by incubation with streptavidin peroxidase. After incubation with the substrate, the reaction in the samples was terminated by adding 1M of H_2_SO_4_ and the optical density was measured at 450 nm in a Thermo Electron Type 1500 Multiskan Spectrum Microplate Reader.

### Statistical Analysis

Statistical analysis was performed using GraphPad Prism software (version v5.0; GraphPad Software Inc., La Jolla, CA, USA). Comparisons between groups were evaluated by non-parametric Kruskal Wallis tests followed by Dunn’s multiple comparisons tests. Differences between groups in the detection of *C.albicans* in fetal blood and gut were analyzed by Fisher’s Exact Test. Data are presented as mean ± standard deviation (SD). Differences were considered statistically significant at p < 0.05.

## Results

### Systemic effects of IA C.albicans administration

Blood cultures for *C.albicans* and plasma levels of IL-6 as shown previously^22^,^28^ are depicted in Table 1.

### C.albicans in the fetal intestine

We evaluated whether *C.albicans* could be detected in the fetal gut tissue by histochemical PAS staining. *C.albicans* was found in fetal gut tissue in 6/7 (86%) animals that were exposed to *C.albicans* for 3 days. In contrast, *C.albicans* was detected only in 2/6 (33%) animals that were exposed to *C.albicans* for 3 days and were treated with fluconazole for 1 day and in 1/5 (20%) animals that were exposed to *C.albicans* for 5 days and were treated with fluconazole for 3 days (Table 1).

**Figure 2:**
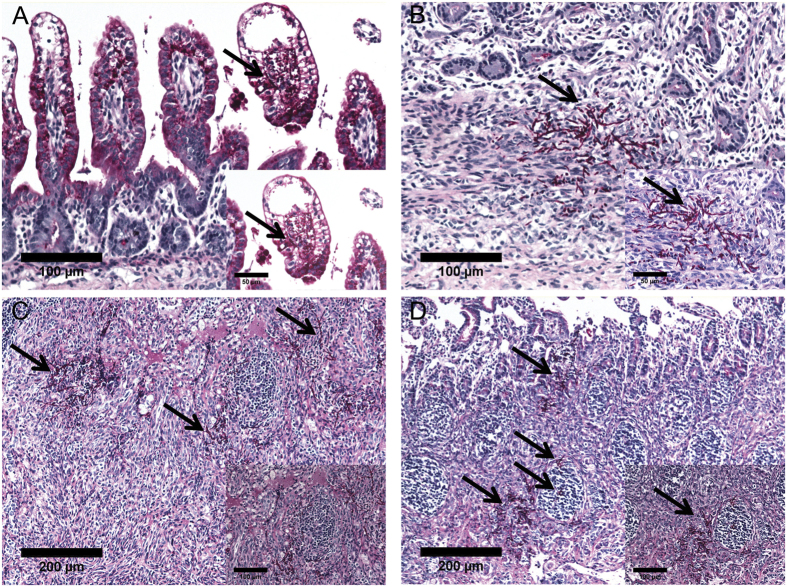
Detection of C.albicans hyphae in the fetal gut tissue. Hyphae of *C.albicans* were detected in the fetal gut at 3 days after IA exposure to *C.albicans*. Hyphae were found in intestinal villi (**A**), lamina propria (**B**), adjacent to (**C**) and inside mucosal Peyer’s patches (**D**) 3 days after of IA exposure to *C.albicans*.

Importantly, *C.albicans* had generated hyphae and invasive growth of *C.albicans* hyphae were found within the intestinal villi (Fig. 2A) and in the lamina propria (Fig. 2B), and particularly in close proximity to or inside Peyer’s patches (PP) (Fig. 2C,D). *C.albicans* was not detected in control animals or animals that had only received fluconazole.

### C.albicans caused gut epithelial injury

**Figure 3:**
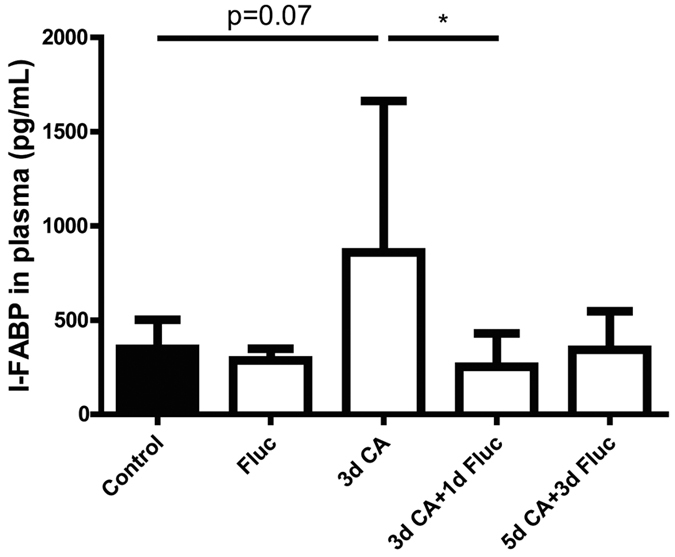
Concentration of I-FABP (pg/mL) in fetal plasma. Intra-amniotic *C.albicans* exposure for 3 days increased the I-FABP levels in the fetal plasma. I-FABP levels were decreased in animals that were treated with fluconazole for 1 day after IA exposure to *C. albicans* Data are presented as mean ± SD. *p < 0.05.

Subsequently, we evaluated whether *C.albicans* exposure caused intestinal epithelial damage by measuring plasma I-FABP, an enterocyte-specific cytosolic protein that is released upon injury^29^,^30^. Significantly increased (p < 0.05) concentration of I-FABP was detected in plasma samples of animals that were infected with *C.albicans* for 3 days when compared to controls (Fig. 3). The elevated I-FABP levels were significantly prevented at 1 day after fluconazole treatment (Fig. 3). I-FABP levels were not changed in the fluconazole only group and 5d *C.albicans* plus 3d fluconazole treatment group when compared to control.

### C.albicans induced infiltration of inflammatory cells in the fetal gut

**Figure 4:**
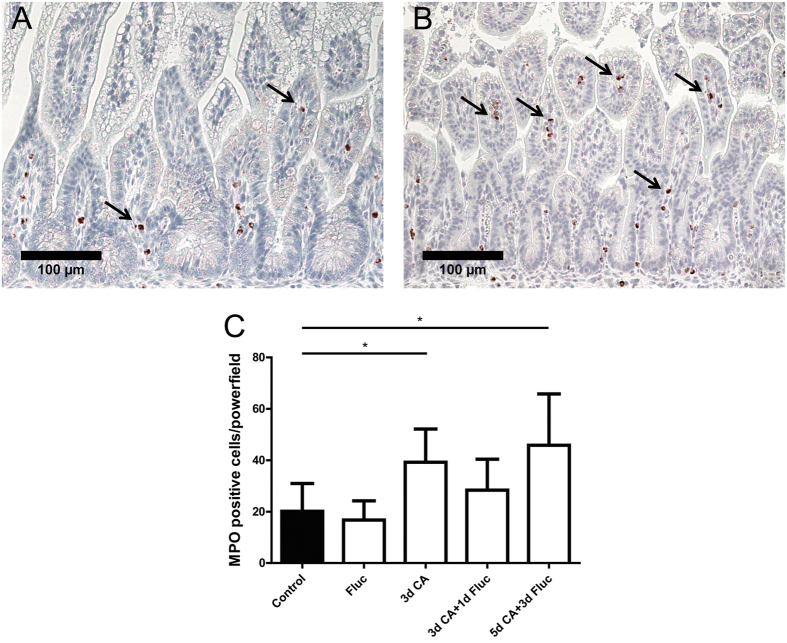
Number of MPO + cells in the fetal ileum. For each experimental group, intestinal sections of (**A**) control and animals that were exposed to *C.albicans* for 3 days (**B**) were stained by immunohistochemistry for MPO. For each experimental group, positive expressing cells of MPO (arrow) were counted and the mean cell counts per high power field per animal is indicated (**C**). Data are presented as mean ± SD. *p < 0.05. MPO, myeloperoxidase.

**Figure 5:**
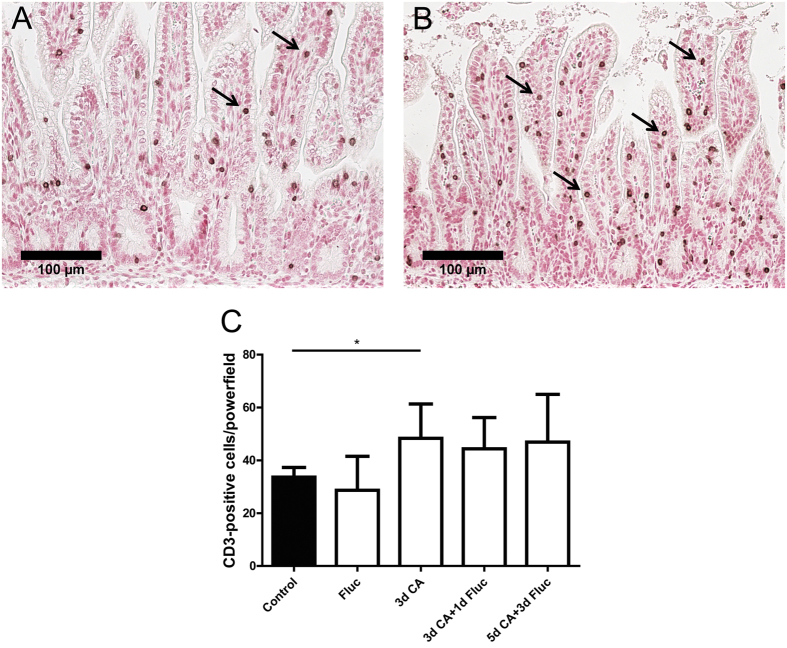
Number of CD3 + cells in the fetal ileum. For each experimental group, intestinal sections of (**A**) control and animals that were exposed to *C.albicans* for 3 days (**B**) were stained by immunohistochemistry for CD3. For each experimental group, positive expressing cells of CD3 (arrow) were counted and the mean cell counts per high power field per animal is indicated (**C**). Data are presented as mean ± SD. *p < 0.05. CD, cluster of differentiation.

We next assessed the accumulation of MPO^+^ and CD3^+^ immune cells in the fetal gut by immunohistochemistry. Significantly increased (p < 0.05) numbers of MPO^+^ cells were found in animals exposed to *C.albicans* for 3 days as well as in animals that were exposed to *C.albicans* for 5 days followed by fluconazole for 3 days when compared with controls (Fig. 4). Similarly, significantly increased (p < 0.05) numbers of CD3^+^ T cells were detected in the fetal gut of animals that were exposed to *C.albicans* for 3 days when compared with controls (Fig. 5). Fluconazole treatment after *C.albicans* exposure did not prevent the accumulation of CD3^+^ lymphocytes in the fetal gut (Fig. 5C).

### C.albicans increased gut mRNA levels of pro-inflammatory cytokines

**Figure 6:**
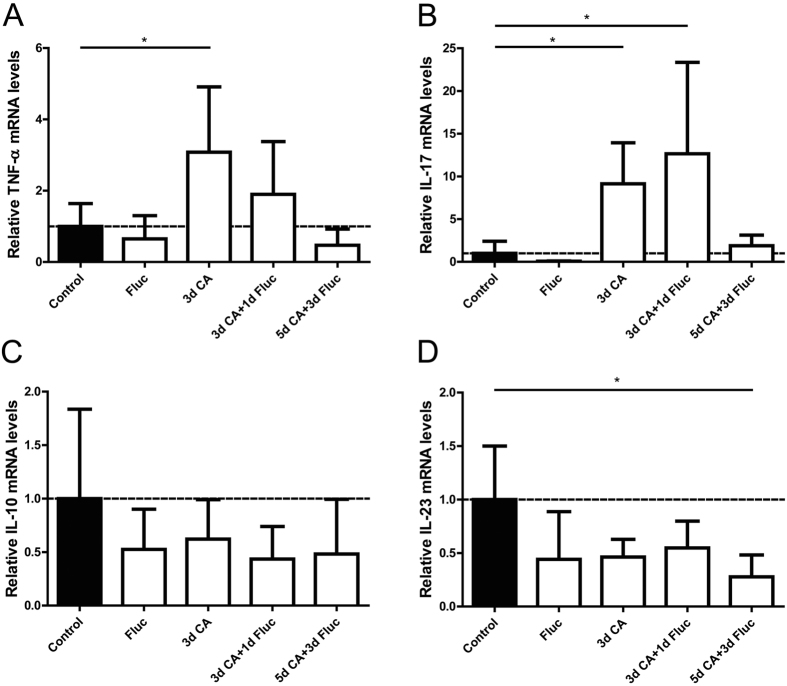
Cytokine mRNA levels in total fetal ileal tissue. The inflammatory cytokines TNF-α (**A**), IL-17 (**B**), IL-10 (**C**) and IL-23 (**D**) were assessed by qPCR and the values for each cytokine were normalized to ovRPS15 rRNA. Data are presented as mean ± SD. *p < 0.05. IL, interleukin; TNF-α, tumor necrosis-α; qPCR, quantitative real time polymerase chain reaction.

We further characterized the inflammatory response by evaluating mRNA levels of pro- and anti-inflammatory cytokines in total gut tissue specimens. *C.albicans* exposure for 3 days significantly increased the intestinal mRNA levels of TNF-α and IL-17 (p < 0.05 for both) when compared to controls (Fig. 6A,B). The elevated TNF-α and IL-17 mRNA levels were not decreased at 1 day after fluconazole treatment. No changes were found in the mRNA levels of animals that were exposed to *C.albicans* for 5 days and treated with fluconazole when compared with controls. Intereukin-10 is known to be produced by the host upon infection with *C.albicans* hyphae and increased levels of IL-10 have been associated with increased susceptibility to candidiasis^31^. The mRNA levels of IL-10 remained unaltered after IA exposure to *C.albicans* with or without fluconazole treatment when compared with controls (Fig. 6C). The intestinal mRNA levels of IL-23 were significantly decreased in animals that were exposed to IA *C.albicans* infection for 5 days and treated with fluconazole compared to controls (Fig. 6D). No changes were detected in gut IL-23 mRNA of the remaining groups.

## Discussion

We have shown that IA exposure of fetal lambs to *C.albicans* resulted in an invasive *C.albicans* infection of the fetal gut tissue associated with epithelial injury and the induction of mucosal inflammation. At this stage, the described intestinal changes were accompanied by fungal translocation and systemic spread of *C.albicans* associated with systemic inflammation. Fluconazole treatment prevented systemic inflammation and *C.albicans* colonization of the fetal gut in the majority of *C.albicans* exposed animals. In addition, administration of fluconazole decreased epithelial injury of the fetal gut without inhibiting intestinal inflammation.

Growth of *C.albicans* in its hyphal form in the gut tissue strongly suggests that the detected mucosal injury is a direct consequence of *C.albicans* infection and translocation through the intestinal epithelial barrier. Invasion of enterocytes is mediated by active penetration of *C.albicans* in which, the hyphal form of *C.albicans* was previously shown to be necessary for the invasive process and the concomitant epithelial injury^32^. Hyphae of *C.albicans* were also in close proximity to PP. Recent *in vitro* experiments have shown that in addition to active penetration of enterocytes, *C.albicans* can translocate the intestinal epithelium via endocytosis by microfold (M) cells. M cells are located at the follicle-associated epithelium (FAE) of the PP^33^. The identification of *C.albicans* hyphae both within intestinal villi and in close proximity to PP suggests that both routes of intestinal translocation might have contributed to the disease observed after IA administration of *C.albicans.*

Epithelial damage as indicated by increased circulatory I-FABP levels following *C.albicans* exposure is an absolute requirement for Candida dissemination to the bloodstream^34^,^35^. Consistently, intestinal colonization and the associated mucosal injury at 3 days after *C.albicans* infection resulted in candidemia. The gastrointestinal tract is considered to be the most frequently organ that is involved in systemic dissemination^36^ and systemic candidiasis is a major contributor to neonatal sepsis^37^. In addition to translocation through the intestinal barrier, *C.albicans* might have breached the mucosa in other mucosal organs exposed to amniotic fluid^38^.

Mucosal invasion of *C.albicans* in the fetal gut by 3 days after infection was associated with infiltration of immune cells and was paralleled by elevated intestinal levels of TNF-α and IL-17. Increased levels of these cytokines were measured in gastrointestinal and oral mucosal surfaces of mice colonized by *C.albicans*^39^,^40^,^41^,^42^. Importantly, induction of TNF-α and IL-17 after *C.albicans* infection has been previously reported to stimulate the recruitment of neutrophils at the site of infection^43^ as detected in our chorioamnionitis model. Neutrophils are believed to be fundamental in the process of phagocytosis against mucosal and disseminated *C.albicans* infection^43^. Additionally, IL-17 is considered to be essential in mucosal immunity against *C.albicans* infection, as increased levels of IL-17 promote inflammation and disease progression^44^. Th17 cells (a hallmark of IL-17 production) are commonly involved in host defense against *C.albicans* in epithelial and mucosal surfaces as they express chemokine receptors which locate them in epithelial and mucosal regions^45^. Evaluating the expression of Th17-specific surface markers would be crucial to identify and important to unravel the precise defense mechanism of fetal intestinal Th17 cells against IA *C.albicans* infection. In addition, establishing the role of intestinal macrophages (a predominant source of TNF-α) against *C.albicans* infection would provide more insight in the process of phagocytosis and the interplay between innate and adaptive immune responses. Unfortunately, considering the current study design and the intrinsic limitations of our model such as the lack of ovine specific reagents, these intestinal immunological responses against IA *C.albicans* infection cannot be studied in detail at this stage. Nevertheless, this animal model was used as the development of the fetal ovine intestine during gestation is similar to human and this large animal model allow us to administer therapeutics, in doses which could potentially be used in humans. Therefore, this model is an appropriate preclinical model to investigate gut complications after *C.albicans-*mediated chorioamnionitis and test the therapeutic potential of IA fluconazole treatment during gestation.

In the same animals, we have previously shown that fluconazole treatment administered 2 days after IA administration of *C.albicans*, temporally prevented systemic inflammation and reduced fetal mortality^22^,^28^. In the present study, fluconazole treatment initiated 2 days after *C.albicans* infection decreased *C.albicans* colonization and epithelial injury of the fetal gut. Remarkably, although circulatory IL-6 levels returned to baseline levels after fluconazole treatment, *C.albicans* infection persisted in the bloodstream of these animals. Since fluconazole treatment after *C.albicans* infection transiently prevented chorioamnion and lung inflammatory changes^22^, we speculated that the decreased IL-6 levels in the fetal plasma following fluconazole treatment were mediated by the lung and/or chorioamnion^46^. Nevertheless, the exact cause of systemic inflammation induced by IA *C.albicans* administration requires further investigation.

The reduction of intestinal *C.albicans* colonization in fluconazole treated animals was not associated with reduced intestinal inflammation. Fluconazole inhibits fungal growth by compromising its cell wall integrity^47^,^48^, which maintains inflammation. In fact the treatment-induced fungal cell destruction might temporarily increase the concentration of immunostimulatory molecules. On the other side, the ongoing inflammatory response is considered essential to eradicate the fungus^49^,^50^.

We have previously shown that fluconazole treatment after *C.albicans* infection was inadequate to eliminate *C.albicans* from the amniotic fluid. Moreover, the inflammatory response in several fetal organs was only temporarily decreased following anti-fungal treatment^22^. These previous results combined with current data indicate that future research is warranted to explore the efficacy of alternative timing, dosing and frequency of fluconazole treatment in the context of intrauterine *C.albicans* infection.

In conclusion, we have shown that IA exposure of fetal lambs to *C.albicans* resulted in gut colonization, mucosal translocation, tissue injury and inflammation. These adverse intestinal consequences are associated with *C.albicans* translocation to the bloodstream with signs of systemic inflammation which was previously shown^22^,^28^. Although we did not assess the postnatal consequences of these findings, gut inflammation and tissue injury in conjunction with systemic inflammation are all pathological features, which are associated with neonatal gastrointestinal pathologies. In line, we have previously reported that chorioamnionitis with a fetal systemic inflammatory response, is strongly associated with NEC^51^. In addition, a single fluconazole treatment prevented systemic inflammation and decreased intestinal injury whereas mucosal inflammation and dissemination of *C.albicans* in the bloodstream remained unaffected. Consequently, future studies should focus on optimizing a fluconazole-based therapeutic strategy to completely protect the fetal gut in the context of *C.albicans*-mediated chorioamnionitis.

## Additional Information

**How to cite this article**: Nikiforou, M. *et al.* Intra-amniotic *Candida albicans* infection induces mucosal injury and inflammation in the ovine fetal intestine. *Sci. Rep.* **6**, 29806; doi: 10.1038/srep29806 (2016).

## Supplementary Material

Supplementary Information

## Figures and Tables

**Table 1:** Detection of *C.albicans* in fetal blood and gut tissue, and IL-6 plasma levels.

	Control	Fluconazole	3d *C.albicans*	3d *C.albicans* + 1d Fluc	5d *C.albicans* + 3d Fluc
Positive *C.albicans* blood culture	0/6 (0%)	0/3 (0%)	5/7 (71%)*OR: 28.6 95% CI (1.12–731.54)	5/6 (83%) *OR: 47.67 95% CI (1.6–1422.69)	3/5 (60%)
Positive PAS staining for Candida in the gut	0/6 (0%)	0/3 (0%)	6/7 (86%)**OR: 56.33 95% CI (1.92–1655)	2/6 (33%)	1/5 (20%)^$^ OR: 0.033 95% CI (0.001–0.68)
IL-6 (ng/mL)	N.D.	N.D.	40 ± 45*^,#^	N.D.	19 ± 29

For the detection of *C.albicans* in blood cultures and gut, the results were analyzed by Fisher’s Exact Test. Data are presented as positive/total number of animals.

For IL-6 results, differences between groups were analyzed by non-parametric Kruskal Wallis tests followed by Dunn’s multiple comparisons test. Data are presented as mean ± SD.

*p < 0.05, **p < 0.01 compared to control. ^#^p < 0.05 compared to 3d *C.albicans* + 1d Fluc group, ^$^p < 0.05 compared to 3d *C.albicans.* CI, confidence interval; Fluc, fluconazole; IL, interleukin; n, number; N.D., not detectable; OR, odds ratio; PAS, Periodic acid–Schiff.
