# Supplementary material for: Intra-amniotic Candida albicans infection induces mucosal injury and inflammation in the ovine fetal intestine
Source: Sci Rep. 2016 Jul 14;6:29806. doi: 10.1038/srep29806 (PMC4944185; doi:10.1038/srep29806)
Supplement: Supplementary Information [file srep29806-s1.doc]

**Intra-amniotic *Candida albicans* infection induces mucosal injury and inflammation in the ovine fetal intestine**

Maria Nikiforou ^1, 2^, Esmee M.R. Jacobs ^2^, Matthew W. Kemp ^3^, Mathias W. Hornef ^4^, Matthew S Payne ^3^, Masatoshi Saito ^3, 5^, John P. Newnham ^3^, Leon E.W. Janssen ^1, 2^, Alan H. Jobe ^3, 6^, Suhas G. Kallapur ^3, 6^, Boris W. Kramer ^1, 2, 7^ and Tim G.A.M Wolfs ^2, 7, 8 *^

1 School for Mental Health and Neuroscience, Maastricht University, Maastricht, the Netherlands

2 Department of Pediatrics, Maastricht University Medical Center, Maastricht, the Netherlands

3 School of Women’s and Infants’ Health, The University of Western Australia, Perth, Western Australia

4 Institute of Medical Microbiology, RWTH University Hospital, Aachen, Germany

5 Division of Perinatal Medicine, Tohoku University Hospital, Sendai, Japan

6 Division of Pulmonary Biology, Cincinnati Children’s Hospital Medical Centre, University of Cincinnati School of Medicine, Cincinnati, OH, USA

7 School of Oncology and Developmental Biology, Maastricht University, Maastricht, the Netherlands

8 Department of Biomedical Engineering, Maastricht University, Maastricht, the Netherlands

**Supplementary Table 1:** Primers used for qPCR.

| ***Primer*** | ***Fw sequence*** | ***Rv sequence*** |
| --- | --- | --- |
| ovRPS15 | CGAGATGGTGGGCAGCAT | GCTTGATTTCCACCTGGTTGA |
| TNFα | GCCGGAATACCTGGACTATGC | CAGGGCGATGATCCCAAAGTAG |
| IL-17 | TGTGAGGGTCAACCTGAACAT | TGATAATCGGTGGGCCTTCTG |
| IL-23 | GGGAAGTGGACAGAGGTTCC | CTGCCTCTCCAATCTGGCTG |
| IL-10 | CATGGGCCTGACATCAAGGA | CGGAGGGTCTTCAGCTTCTC |

Abbreviations: Fw: forward; IL: interleukin; qPCR: quantitative real time polymerase chain reaction; Rv: reverse.
